# Does attention bias modification training impact on task performance in the context of pain: An experimental study in healthy participants

**DOI:** 10.1371/journal.pone.0200629

**Published:** 2018-07-18

**Authors:** Dimitri M. L. Van Ryckeghem, Stefaan Van Damme, Tine Vervoort

**Affiliations:** 1 Institute for Health and Behaviour, INSIDE, University of Luxembourg, Esch-sur-Alzette, Luxembourg; 2 Department of Experimental Clinical and Health Psychology, Ghent University, Ghent, Belgium; University of Rome, ITALY

## Abstract

Attention has been theorized to play a key role in the experience of pain and associated task interference. Training attention *away* from pain via attention bias modification (ABM) training techniques has been proposed to improve pain-related outcomes, but evidence is inconsistent. In an experimental study, we investigated the impact of a single session ABM training -using a visual probe paradigm with idiosyncratic pain words- on cold pressor test (CPT) pain experience and task interference by pain. Fifty-eight healthy volunteers were randomly assigned to an ABM training group (*N* = 28; attending away from pain) and a sham training group (*N* = 30; no training direction). At pre-training, participants performed a baseline Random-Interval-Repetition (RIR) task and the CPT. Participants reported on sensations they experienced during the baseline CPT. Relevant descriptors were integrated in the visual probe paradigm during the training phase. At post-training, participants completed the RIR task again while experiencing CPT pain. Participants also reported on the extent they attended to the pain and the intensity/unpleasantness of the pain. Results indicated that, in contrast with our hypotheses, ABM training did also not reduce task interference due to CPT pain. Furthermore, ABM training did not change self-reported attending to CPT pain. Finally, ABM training did not reduce CPT pain intensity or pain unpleasantness. Overall, the current study provides no support for the effectiveness of a single session ABM training in improving pain-related outcomes. Future research addressing the conditions under which ABM training improves or fails to improve pain-related outcomes is warranted.

## Introduction

Attention plays a pivotal role in the experience of pain and its impact upon task performance [1,2,3,4]. In particular, research amongst healthy volunteers and chronic pain patients has shown that attention bias toward pain-related information—i.e., the tendency to select pain information over non-pain information—increases the experience of pain [5,6] and the level of interference by pain with ongoing activities [7,8,9]. Given the key role of attention bias, attempts have been undertaken to investigate whether pain-related attention biases can be reduced using Attention Bias Modification (ABM) training, and whether ABM training affects pain and/or pain-related outcomes. ABM training typically consists of a computerized training protocol in which participants are trained to attend away from pain-related stimuli and towards neutral stimuli [10,11,12,6]. As yet, research investigating the effectiveness of ABM is still in its infancy. Early evidence has indicated that ABM can be effective in improving certain pain-related outcomes. In particular, experimental research in healthy adults indicated that a single ABM training session away from pain increased CPT pain outcomes (e.g., pain threshold [10, 13], pain intensity [14]). However, the effects of single session ABM training on pain-related outcomes are inconsistent and recent findings of a single session ABM training in healthy adults failed to replicate the positive effects of ABM training away from pain [15]. Similar inconsistent effects of ABM training on pain-related outcomes have been reported in chronic pain patients (See [16,17], but see [11]).

Laboratory studies optimizing ABM training procedures are needed to address this inconsistency in findings as it may relate to methodological differences and limitations of the applied ABM training approaches. Most often, a standardized set of stimuli is chosen to assess attention bias as well as to train attention away from pain-related information. The use of *idiosyncratic* stimuli is however preferable as it ensures that people are trained away from stimuli that activate their personal pain schemata [18]. Furthermore, all, except one [10], previous single session ABM training studies have compared training *attention away* from pain information with training *attention towards* pain information [14,13,15]. Comparing the training of attention away from pain-related information with the training of attention towards pain-related information does however not allow to draw conclusions on the isolated effect of both training conditions. To do so, there is need for experimental studies including a *sham condition* (i.e., a condition with no training direction) to isolate the effect of training attention away from pain. Finally, researchers have focused on the effect of ABM training on the experience of pain, operationalized in a variety of ways, such as pain intensity, pain threshold and pain tolerance. Yet, theoretical advances have suggested that attentional bias may not easily amplify the experience of pain. Instead, the presence of pain may result in more task interference in those who have an increased attention bias towards pain-related information [9,19]. Following this reasoning, the effects of ABM training should be investigated in a context of competition for attention (i.e., competition between pain and a competing task). Available studies have often looked at the impact of ABM training on pain outcomes in isolation of competing goals. Hereby, people need to report on the pain threshold or pain tolerance (which requires attention for the pain sensation) [7,20,21]. At current, none of the available laboratory studies has examined the impact of ABM training upon *task interference by pain*.

In the present study, we aimed to investigate the impact of a single ABM training session, using idiosyncratically selected pain words, on the experience of CPT pain and, especially, its interference effect on a competing task. We hypothesized that, in comparison with sham training, ABM training away from pain stimuli would (a) reduce attention bias for pain-related information, (b) diminish task interference due to CPT pain (primary outcome), (c) decrease self-reported CPT pain intensity/unpleasantness when performing a competing task as well as self-reported attending to CPT pain (secondary outcomes).

## Materials and methods

### Participants

Participants were undergraduate students from Ghent University with normal or corrected-to-normal vision, who received course credits for participation. Exclusion criteria were a history of seizures, cardiovascular diseases, frostbite, cuts, sores or fractures on the left hand to be immersed, or Raynaud disease [22]. Participants were also excluded if (1) they reported a history of chronic pain or (2) they reported pain intensity at moment of testing >3 on a VAS scale (0 = no pain; 10 = worst possible pain; [23]). Furthermore, proficiency in the Dutch language was required (evaluated by the experimenter during the debriefing phase at the end of the experiment). Based upon the findings of McGowan and colleagues (2009), a power analysis indicated that 27 participants would be needed per group to achieve 80% power (*α* = .05). To have sufficient power we aimed for 60 participants in this study. Experimental procedures were approved by the Ethics Committee of the Faculty of Psychology and Educational Sciences of Ghent University, and written informed consent was obtained from participants.

### Task stimuli

The word list contained 20 sensations that one could experience during the CPT and 20 matched neutral words (see S1 Table for a list of sensations). The pain words were drawn from the McGill pain inventory and previous research assessing the experience of CPT pain in the Ghent Heath Psychology lab (e.g., [24]). Neutral word stimuli were Dutch words, which were matched for length and frequency in Dutch language using Wordgen 1, a computer program that uses the CELEX and Lexique lexical databases for word selection [25]. For the visual probe task, a set of six idiosyncratic words was selected per participant. This selection was based upon the personal relevance of the felt sensation during the CPT assessed at baseline (i.e., in advance of the visual probe task). The personal relevance of each sensation was probed via a single question: “The sensation you had during the CPT in your hand/dust was … (sensation; 0 = not at all; 10 = very much)”. The six words with the highest ratings were selected and included in the visual probe task. If more than six words were possible to select (i.e., with a similar high rating), a random selection was taken.

### Experimental tasks

#### ABM and sham training

**Fig 1:**
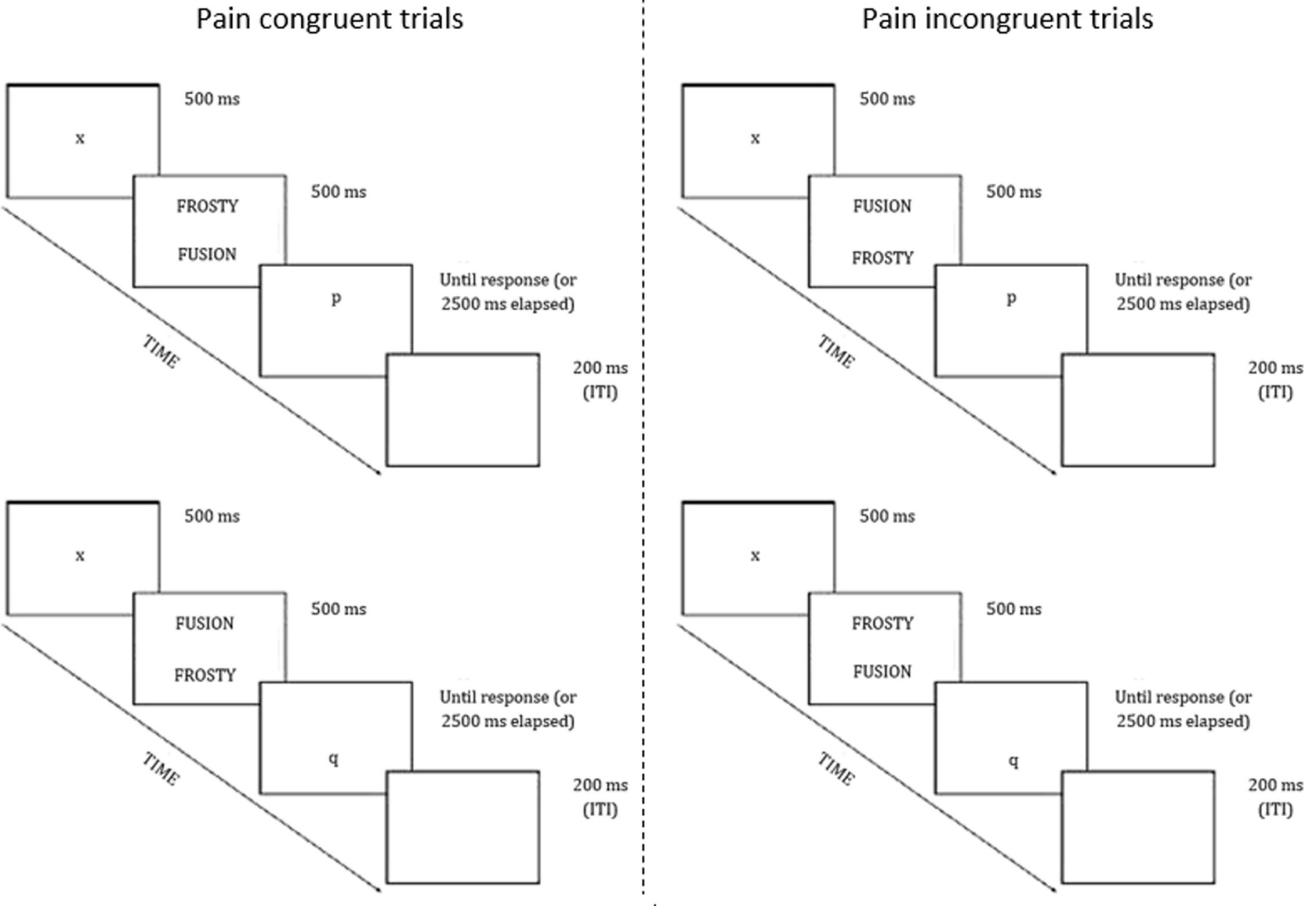
Trial types of the ABM and sham training paradigm.

The ABM and sham training were presented via Inquisit Millisecond software (Inquisit 3; Seattle, WA: Millisecond Software) on a 60-Hz, 19-inch color monitor. ABM and sham training were delivered using modified versions of the visual probe paradigm (e.g., [6,12]). During ABM and sham training, stimuli were presented against a black background. Each trial began with a 500 ms presentation of a white fixation cross in the middle of the screen. Then, one stimulus pair comprising a pain word and a neutral word appeared and remained visible for 500 ms. The visual angle of the word stimuli was 7.13° above or below the center of the screen. One stimulus was presented above and one below the fixation cross. Immediately after the offset of these two words, a letter ‘p’ or ‘q’ (i.e., probe) appeared at one of the word locations. For all participants, the visual probe paradigm started with a baseline phase. During the baseline phase, the probe appeared equally often in the location of the pain word as in the location of the neutral word, and word pairs were randomly presented in each of the four possible combinations (probe up/ pain stimulus up; probe up/ pain stimulus down; probe down/ pain stimulus down; probe down/ pain stimulus up) (see Fig 1). The baseline phase was immediately followed by the training phase (sham or ABM). Participants received no information concerning the percentage of trials in which the pain word was followed by the dot; neither did they receive information concerning a possible change in the percentage of trials in which the pain word was followed by the dot during the task. During ABM training, the probe appeared in 87.5% of the trials at the previous location of the neutral stimulus and 12.5% of the trials at the previous location of the pain stimulus (see [26,27] for a similar approach). This set-up allowed calculating the change in attentional bias by comparing the attention bias index measured before the training and the attention bias index measured during the last block of the training phase, without adding a post-training phase where half of the trials are again pain congruent. The presence of such post-training phase has been suggested to dilute ABM training effects [15]. During the sham training, the probe appeared equally often at the location of the pain word as at the location of the neutral word. Word pairs were randomly presented in each of the four possible combinations. In both conditions, participants had to indicate whether the probe was a ‘p’ or a ‘q’ by pressing the corresponding button on the keyboard (AZERTY) as accurately and as quickly as possible. The ‘q’ key was pressed with the left index finger and the ‘p’ key was pressed with the right index finger. The trial ended (i.e., disappearance of the probe) immediately after each response, or when 2500 ms elapsed without response. When a participant responded erroneously or answered to late, the term ‘error’ appeared on the screen for 200 ms. In order to ensure that participants maintained gaze at the middle of the screen at the start of each trial, a number of digit trials were presented (see e.g., [28,29]. In these trials, the fixation cross was followed by a random digit between one and nine for a duration of 150 ms. Participants were instructed to type the number on the keyboard. The inter-trial interval was 200 ms after test trials, or 1000 ms after digit trials (i.e., to allow participants to replace their fingers on the ‘p’ and ‘q’ buttons). In the context of the current study, congruent trials were those where the probe was presented at the same location as the pain word. Incongruent trials were those where the probe was presented at the opposite location as the pain word. The baseline phase consisted of 105 trials (48 congruent trials, 48 incongruent trials, 9 digit trials). The training session consisted of four blocks each consisting of 105 training trials (sham condition: 48 congruent trials, 48 incongruent trials, 9 digit trials; ABM condition: 12 congruent trials, 84 incongruent trials, 9 digit trials). Stimuli were presented in a randomized order across trials and participants, and trials were intermixed and randomly presented in four blocks. Participants received the possibility to have a break in between each phase/block (i.e., 4 breaks).

#### RIR task

The RIR-task is an attention-demanding tone-detection task, which requires executive processing [30]. Previous research has shown that performance of the RIR task was reduced by the experience of pain (e.g., [31,32]). Participants are required to respond as quickly and correctly as possible to tones (tone duration = 150 ms; tone pitch = 750 Hz; inter-stimulus interval 900 and 1500 ms) generated by a computer (ASUS L2000). Tones are presented at random stimulus interval through headphones (Sony MDR-V150). In this study, the total RIR-task duration was 1 minute during which 51 tones are presented. Responses were made by pressing a button pressing device, held in the right hand. As such, the performance of the RIR task was compatible with the performance of the CPT (see below). Task performance was assessed by reaction times (RT), standard deviations (SD) and errors [33]. RTs faster than 100 ms were considered anticipations and omitted from the analyses. Outliers (RTs > 3 *SD* above the individual mean) and omissions were also removed. Errors were calculated by summing anticipations and omissions [33].

#### CPT pain induction

The cold pressor apparatus consisted of a metallic container (Techne B-26 with TE-10D, 530 325 172 mm) filled with water retained at 10°C (±0.1) with a Techne Dip Cooler RU-200 and kept circulated using a water pump. A temperature of 10° was selected based on theoretical considerations and previous research using a similar water temperature [22,24]. Theoretically, it is assumed that attention strategies are less effective when pain is highly intense [21,34,35]. Increasing the temperature from 5° (e.g., [13]) to 10°—which results in a decrease of pain intensity and pain unpleasantness (e.g., [36])—was therefore expected to increase the likelihood for ABM training to impact on pain outcomes. Furthermore, increasing the temperature reduces the amount of people who are not able to immerse their left hand in the cold water container for the fixed duration of one minute [24]. Another container, filled with water at room temperature, was used to standardize hand temperature before immersion of the hand in the cold water container (e.g., [33]). Immersion time in the water at room temperature was one minute.

### Self-report measures

Depressive mood, anxiety and stress was assessed using the Depression Anxiety Stress Scales (DASS; [37]). Each sub-scale contains 14 items (e.g. “I found it hard to wind down”) on which participants indicate how they felt during the past week. Items are assessed using a 4-point Likert scale ranging from zero (“did not apply to me at all”) to three (“applied to me very much, or most of the time”). In the present study, Cronbach’s alpha for the depression, anxiety and stress subscales were respectively .91, .90 and .90.

Pain catastrophizing was assessed using the Pain Catastrophizing Scale (PCS; [38]). The PCS contains 13 items that measure catastrophic thoughts about pain in both clinical and non-clinical samples. Participants reflect on past painful experiences and indicate on a 5-point scale ranging from zero (“not at all”) to four (“always”) the degree to which they experience each of the 13 thoughts or feelings during the experience of a pain (i.e. “When I’m in pain it’s terrible and I think it’s never going to get any better”). Research has shown that the PCS is valid and reliable [39]. In the present study, Cronbach’s alpha of the total score was .87.

Trait anxiety was assessed by means of the State-Trait Anxiety Inventory-Trait version (STAI-T; [40,41]). This questionnaire consists of 20 items in which people are asked to report their feelings in general (e.g., I feel happy) using a four-point Likert scale. Scores may vary between 20 and 80. This questionnaire showed a good reliability and validity [42,43]. In the present study, Cronbach’s alpha of the STAI-T was .93.

Attention Control was assessed by means of the Attention Control Scale (ACS; [44]). The ACS consists of 20 items and yields a total score that can range from 20 to 80, with higher scores indicating good attention control. The ACS consists of two subscales: attention focusing (e.g., “my concentration is good even if there is music in the room around me”) and attention shifting (e.g., “It is easy for me to alternate between two different tasks”). The ACS has shown both good reliability and predictive utility [44]. Cronbach alpha in this study was .74.

### Self-reported pain outcomes during CPT

Attention to pain during the CPT was measured with a single item (How much attention have you paid to the pain during the immersion of your hand in the cold water?) [33]. Participants rated the amount of attention they paid to the pain using a 11-point scale (0 = ‘‘no attention at all”; 10 = ‘‘a lot of attention”). Participants reported on experienced sensory and affective pain experience. Sensory pain was assessed by asking participants about experienced pain intensity using two items. Specifically, participants indicated the worst pain and the pain just before the end of the immersion in the cold water on a 11-point scale (0 = ‘‘no pain”; 10 = ‘‘the worst imaginable pain”) [33,45]. A total pain intensity score was computed as the average of both items (range 0–10). Affective pain was indexed by asking participants about experienced pain unpleasantness assessed by means of three items. Specifically, participants indicated how unpleasant the experience was and how anxious and tense they felt during immersion on a 11-point scale (0 = ‘‘pleasant/relaxed/not anxious”; 10 = ‘‘unpleasant/tense/very anxious). A total pain unpleasantness score was calculated by averaging the score of the three items (range 0–10) [33]. Cronbach’s alpha for the pain intensity and pain unpleasantness was .79 (pre-training) and .79 (post-training) and .73 (pre-training) and .78 (post-training), respectively.

### Procedure

Upon arrival in the laboratory, participants received information concerning the experiment session and were told that the aim of the experiment was to investigate “how an emotional event influences cognitive functioning.”. Participants were informed that they would perform a one-minute during CPT twice, once at the beginning and once at the end of the experiment session. In between of the CPTs, they would perform two reaction time tasks (i.e., RIR and visual probe paradigm). In doing so, participants were unaware of the training aspect of the study and anticipated CPT pain during the performance of the visual probe paradigm. All instructions were equal for both conditions. Next, participants filled out a questionnaire battery, containing demographic questions (e.g., Sex, Age, Pain experience at this moment), PCS, ACS, DASS and STAI. Next participants performed a one-minute RIR task (practice phase), to minimize learning effects between RIR-task performance before the attention training phase and after the attention training phase later on. This was followed by the RIR task without CPT (baseline RIR). Next participants performed the one-minute CPT without the RIR-task (baseline CPT). Following the CPT, participants reported on pain outcomes (attention for pain, pain intensity and pain unpleasantness during the baseline CPT). Then, the pain words, i.e., 20 possible sensations, were rated on personal relevance for their pain experience during the baseline CPT. Then, the ABM training phase took place; participants were randomly assigned either to the ABM or the sham condition using a computerized random number generator (www.random.org/). After the training phase, participants performed the RIR task during the CPT for one minute and afterwards reported on all pain outcomes during the second CPT. All participants completed the CPT twice. Afterwards participants were debriefed and thanked for their participation. The entire duration of the experiment was approximately 60 minutes. Participants and experimenter were both blinded to the experiment condition to which participants were assigned.

### Data analyses

Statistical analyses were performed with SPSS statistical software, version 24.0 for Windows (SPSS Inc., Chicago, IL). To address training effects, an attention bias index was calculated by subtracting mean reaction times of congruent trials from mean reaction times of incongruent trials of the visual probe task for the baseline and last training block. Next, a repeated measures analysis of variance (ANOVA) with Phase (baseline vs last training block) and Pain congruency (congruent vs incongruent trials) as within-subject factor and Group (ABM vs sham) as between-group factor was conducted for participants’ attention bias index. To address the impact of ABM training on pain-related outcomes, an analysis of covariance (ANCOVA) was performed with training condition (ABM vs sham) as between-subject variable and baseline assessment of the outcome variable as a covariate for each of the investigated outcome variables; i.e., RIR task performance, pain intensity, pain unpleasantness and attention for pain. This method of analyzing is more powerful and precise than using repeated measures ANOVA in a randomized pre-post design [31]. To explore moderation effects of attention bias index change, attentional control, pain catastrophizing and state and trait anxiety, all analyses were repeated while including the main effect of the attention bias index change score, ACS, PCS, DASS-A and STAI-T and their interactions with training condition as a covariate in separate analyses. All continuous variables entered as covariate in the ANCOVA were centered. For all ad-hoc analyses, the cut-off for statistical significance was set at *p* < 0.05, whereas for all post-hoc analyses (i.e., exploration of moderation effects) a Bonferroni correction was applied resulting in a cut-off for statistical significance of *p* < 0.01. For all analyses, effect sizes were reported using the partial eta squared index (*η*_p_^2^) [46].

## Results

### Participants’ descriptive statistics

The participants were 62 university students of which four were excluded. Three of these participants reported a pain score larger than 3/10 on the NRS assessing pain at the start of the experiment session. For one participant data of the dot-probe task were not registered. The mean age of the final dataset of 58 participants was 18.64 years (*SD* = 1.53; range 17–24 years). The majority of the sample (i.e., 47) was female (81%). Participants assigned to the ABM group and the sham training group did not differ in terms of age, gender, anxiety, depression, catastrophizing, stress and level of attentional control measured at baseline (see Table 1).

**Table 1:** Demographics and baseline characteristics for both training groups.

	Sham (*n* = 30)	ABM (*n* = 28)	Group difference statistic
Sex (females/males)	25/5	22/ 6	χ^2^_(1)_ = 0.21, *ns*
Age (*M* (*SD*))	18.50 (1.48)	18.79 (1.60)	*t*_(56)_ = 0.71, *ns*
Pain intensity (*M* (*SD*))	0.57 (.97)	0.43 (.69)	*t*_(56)_ = 0.62, *ns*
PCS (*M* (*SD*))	17.23 (6.97)	17.04 (8.28)	*t*_(56)_ = 0.10, *ns*
DASS-A (*M* (*SD*))	5.47 (6.61)	4.25 (4.28)	*t*_(56)_ = 0.83, *ns*
DASS-D (*M* (*SD*))	5.00 (5.99)	4.04 (4.91)	*t*_(56)_ = 0.67, *ns*
DASS-S (*M* (*SD*))	10.77 (6.96)	8.57 (6.57)	*t*_(56)_ = 1.23, *ns*
STAI-T (*M* (*SD*))	42.37 (10.46)	38.11 (8.78)	*t*_(56)_) = 1.67, *ns*
ACS (*M* (*SD*))	49.80 (5.08)	51.57 (7.56)	*t*_(56)_ = 1.05, *ns*

### Attention bias outcomes

**Fig 2:**
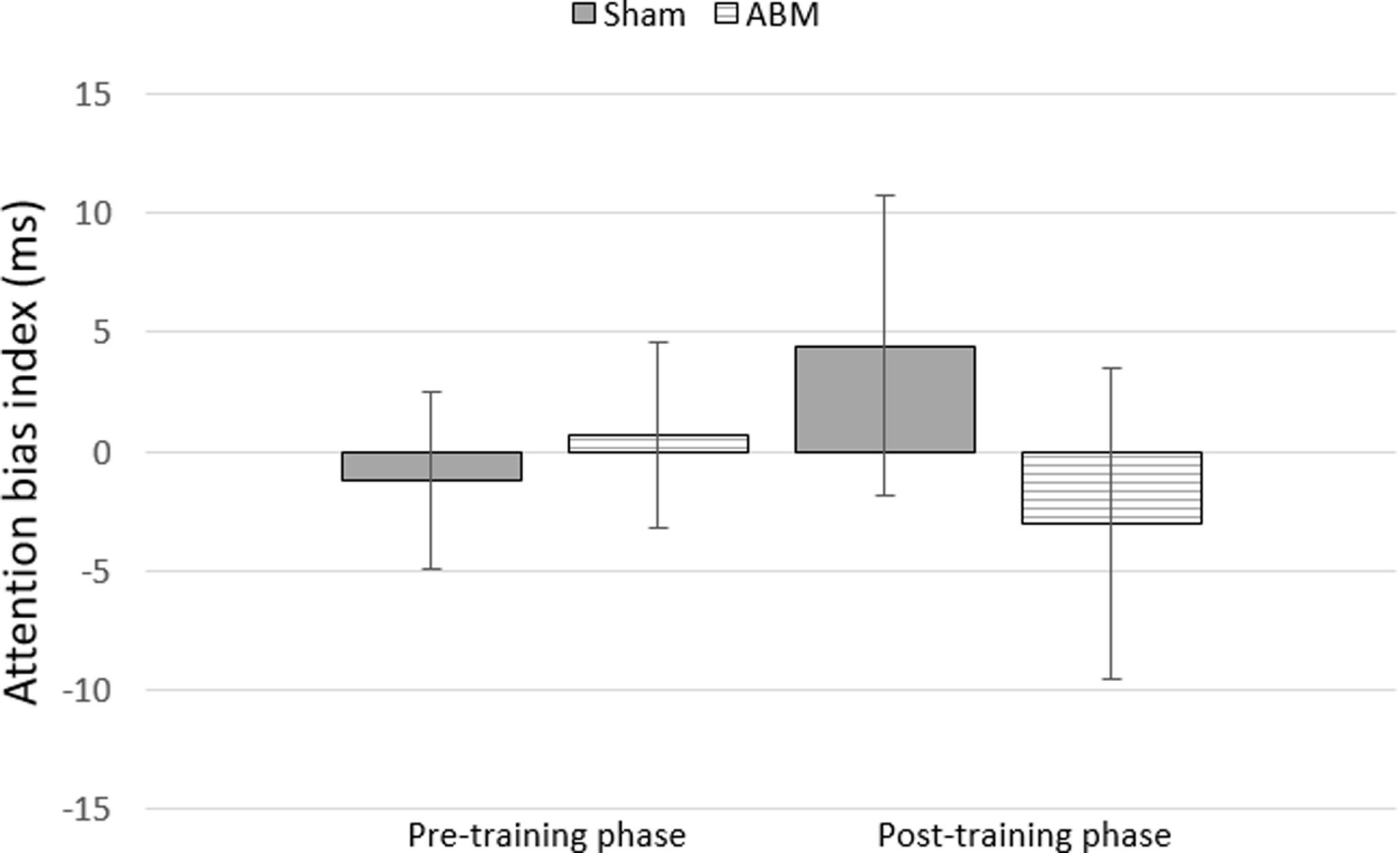
Change in attention bias index per training group.

Before performing reaction time (RT) analyses on the attention bias index, errors and omissions (6.1%) and outliers (2.4%) were removed. Data with response latencies shorter than 200 ms or longer than 1000 ms were considered outliers and excluded from the analyses (e.g., [13]). Analyses were performed on 91.6% of the visual probe RT data. Next, a 2 (Phase: baseline vs last training block) x 2 (Pain congruency: pain congruent vs pain incongruent) x 2 (Group: ABM vs sham) repeated measures ANOVA was performed on the attention bias index. Results showed a main effect of Phase (*F*_(1,56)_ = 11.61, *p* < .001, *η*_p_^2^ = 0.17) indicating that participants were faster in the last training block than at baseline. No main effect of Congruency (*F*_(1,56)_ = 0.01, *ns*, *η*_p_^2^ = 0.00) or Group (*F*_(1, 56)_ = 0.08, *ns*, *η*_p_^2^ = 0.00) was found. Also the hypothesized Phase x Pain congruency x Group interaction effect proved to be non-significant (*F*_(1,56)_ = 0.68, *ns*, *η*_p_^2^ = 0.01), indicating that the training did not significantly change participants’ attention bias for pain-related information. The mean attention bias index for each phase for both training groups is presented in Fig 2.

### Pain-related outcomes

#### Task performance

The effect of training on RIR task performance was investigated by means of three ANCOVAs. A first ANCOVA with RIR mean latency as dependent variable, training condition (ABM vs sham) as between-subject variable and baseline RIR mean latency (i.e. for pre-training RIR; mean centered) as a covariate revealed a main effect of baseline RIR mean latency (*F*_(1,55)_ = 33.87, *p* < .001, *η*_p_^2^ = 0.38), but not of training condition (*F*_(1,55)_ = 0.12, *η*_p_^2^ = 0.00) in explaining post-training RIR mean latency during CPT. A second ANCOVA with RIR errors as dependent variable, training condition (ABM vs sham) as between-subject variable and baseline RIR errors (i.e., for pre-training RIR; mean centered) as a covariate revealed no main effect of training condition (*F*_(1,55)_ = 0.00, ns, *η*_p_^2^ = 0.00) or baseline RIR errors (*F*_(1,55)_ = 3.04, ns, *η*_p_^2^ = 0.05) in explaining post-training RIR errors during CPT. A third ANCOVA with RIR *SD* as dependent variable, training condition (ABM vs sham) as between-subject variable and baseline RIR *SD* (i.e. for pre-training RIR; mean centered) as a covariate also revealed no main effect of training condition (*F*_(1,55)_ = 0.00, ns, *η*_p_^2^ = 0.00) or baseline RIR *SD* (*F*_(1,55)_ = 1.07, ns, *η*_p_^2^ = 0.02) in explaining post-training RIR *SD* during CPT.

Additional analyses with each task performance index separately as dependent variable showed no additional significant main effects of attention bias index change, pain catastrophizing, attentional control, state and trait anxiety or interaction effects between these variables and Group; RIR mean (All *F* < 3.35, ns), RIR errors (All *F* < 6.06, *p* > .01), and RIR SD (All *F* < 3.46, ns). Means, percentage errors and *SD*s for RIR task performance measures per training group are shown in Table 2.

**Table 2:** Means (M) and standard deviation (SD) for pain-related outcomes separated for training group and test phase.

	Sham condition (*n* = 30)			ABM condition (*n* = 28)		
	Pre-training *M* (*SD*)	Post-training *M* (*SD*)	*d* (CI)	Pre-training *M* (*SD*)	Post-training *M* (*SD*)	*d* (CI)
Pain intensity	6.10 (1.77)	6.53 (1.89)	0.23 (0.03:0.43)	6.09 (1.92)	6.59 (2.06)	0.25 (0.07:0.43)
Pain unpleasantness	5.33 (1.66)	5.41 (1.72)	0.05 (-0.21:0.30)	5.77 (2.00)	5.56 (2.06)	-0.10 (-0.36:0.15)
Attention for pain	4.90 (2.62)	4.27 (2.05)	-0.26 (-0.62:0.10)	5.96 (2.65)	5.21 (2.10)	-0.31 (-0.64:0.03)
*M* latency RIR task	179.89 (41.65)	197.35 (64.43)	0.29 (0.01:0.58)	183.77 (41.64)	196.54 (39.12)	0.32 (-0.05:0.69)
*SD* latency RIR task	39.64 (19.85)	49.61 (33.21)	0.36 (-0.11:0.83)	40.27 (26.98)	49.26 (29.29)	0.32 (-0.19:0.83)
% Errors RIR task	1.44 (1.78)	3.92 (4.02)	0.78 (0.25:1.30)	1.82 (3.02)	4.13 (4.35)	0.61 (0.12:1.10)

#### Self-reported pain experience

The effect of training on experienced pain intensity was investigated using an ANCOVA with training condition (ABM vs sham) as between-subject variable and baseline pain intensity (i.e., during pre-training CPT; mean centered) as a covariate. Results revealed a main effect of baseline pain intensity (*F*_(1,55)_ = 171.48, *p* < .001, *η*_p_^2^ = 0.76), indicating that higher baseline pain intensity related to higher pain intensity during the second CPT. No effect was found for training condition (*F*_(1,55)_ = 0.07, ns, *η*_p_^2^ = 0.00) in explaining post-training pain intensity. Additional analyses showed no significant main effects of attention bias index change, pain catastrophizing, attentional control, state and trait anxiety or interaction effects between these variables and Group (All *F* < 2.60, ns).

The effect of training on pain unpleasantness was investigated using an ANCOVA with training condition (ABM vs sham) as between-subject variable and baseline pain unpleasantness (i.e., during pre-training CPT; mean centered) as a covariate. Results revealed a main effect of baseline pain unpleasantness (*F*_(1,55)_ = 74.83, *p* < .001, *η*_p_^2^ = 0.58), indicating that higher baseline pain unpleasantness related to higher pain unpleasantness during the second CPT. However, again, no main effect of training condition (*F*_(1,55)_ = 0.36, ns, *η*_p_^2^ = 0.01) in explaining post-training pain unpleasantness was found. Additional analyses showed a significant main effect of pain catastrophizing (*F*_(1,55)_ = 8.07, *p* < .01, *η*_p_^2^ = 0.13), indicating that higher levels of pain catastrophizing were associated with higher levels of self-reported pain unpleasantness. No other main effects were found for attention bias index change, pain catastrophizing, attentional control, state and trait anxiety or interaction effects between these variables and Group (All *F* < 4.57, *p* >.01). Means and *SD*s for pain experience measures per training group are shown in Table 2.

#### Self-reported attention for pain

The effect of training on attention for pain was investigated using an ANCOVA with training condition (ABM vs sham) as between-subject variable and baseline attention for pain (i.e., during pre-training CPT; mean centered) as a covariate. Results revealed a main effect of baseline attention for pain (*F*_(1,55)_ = 25.56, *p* < .001, *η*_p_^2^ = 0.32), indicating that higher baseline attention for pain related to higher attention for pain during the second CPT. Again no effect of training condition (*F*_(1,55)_ = 1.05, ns, *η*_p_^2^ = 0.02) was found in explaining post-training attention for pain. Additional analyses showed no significant main effects of attention bias index change, pain catastrophizing, attentional control, state and trait anxiety or interaction effects between these variables and Group (All *F* < 4.73, *p* > .01). Means and *SD*s for attention for pain measures per training group are shown in Table 2.

## Discussion

The primary aim of the current study was to investigate the impact of a single ABM training session on pain-related task interference. In addition, we investigated the impact of a single ABM training session on participants’ pain experience when performing a competing task in a controlled laboratory context. Importantly, these aims were addressed, while optimizing the stimulus content used for the ABM training procedure. In particular, we used idiosyncratic pain words instead of a standard set of pain words. The use of stimulus content that activates participants’ personal pain schemata is considered essential in effectively measuring and manipulating attention bias for pain [18]. Furthermore, we used parameters that have shown to have the largest impact on pain experience in previous ABM research (i.e., pain words instead of pain pictures [13]); stimulus presentation time of 500 ms [10,13]). Results of the current study can be readily summarised. First, the ABM training did not significantly change participants’ level of attention bias for pain. Furthermore, while pain was found to interfere with task performance, ABM training did not result in better task performance when experiencing pain. Finally, and in contrast to earlier findings showing that ABM training affects self-reported pain experience, no evidence was found for the impact of ABM training upon pain experience.

The current findings are in contrast with earlier research examining the effect of a single session ABM training on experimental pain experience. We briefly review prior experimental studies investigating the effect of a single ABM training session on acute pain outcomes to identify differences in methodology and setting that may explain the contrasting findings. McGowan and colleagues, who were the first to investigate the effects of a single ABM training session on pain, found that training attention away from pain words changed the attention bias index in the expected direction and resulted in an increased pain threshold and reduced pain experience at 30s CPT immersion compared to training attention towards pain words [14]. No training effect was found for pain tolerance. In a follow-up study, Sharpe and colleagues found again that single-session ABM training changed the attention bias index in the predicted direction [13]. Furthermore, participants who received training away from painful stimuli had a higher pain threshold than those who were trained to attend towards painful stimuli. No effects of ABM training were found on pain experience 30s after CPT immersion and pain tolerance. More recently, Todd and colleagues found, in contrast to previous studies, that a single session of ABM training towards affective pain words resulted in a higher pain threshold compared to training attention away from pain words [15]. People trained towards affective pain words reported also higher levels of distress at tolerance. No effect was found of training with sensory pain words. In this study, ABM training did not change the attention bias index. Finally, Bowler and colleagues investigated the effects of a single ABM training session away from pain and compared its effects on CPT outcomes with a sham training [10]. Bowler and colleagues found no effect of ABM training on attention bias index. A positive effect of ABM training away from pain-related words was found on pain threshold and pain tolerance. Effects on pain experience at 30s following immersion did not reach significance. Furthermore, these findings were only true when pain stimuli were presented for 500ms. None of the pain outcomes was modified when pain stimuli were presented for 1250ms.

This brief overview points at a number of reasons that may explain why the current findings differ from earlier research findings. First, only one previous study has compared training attention away from pain with a sham training [10]. All other studies compared training attention *away* from pain with a control condition in which attention was trained *towards* pain stimuli [13,14,15]. As such, it may well be that the differences between training conditions were mainly driven by the condition in which attention was trained towards pain. Although the comparison of ABM training away from pain with ABM training towards pain may enlarge the difference between both training conditions, future research should include a sham training condition to enable the isolation of the (positive) effect of ABM training away from pain on pain-related outcomes. Second, our null-findings may be due to slight differences in training protocol. The current ABM training away from pain was done by presenting the probe in 87.5% of the trials at the location of the neutral stimulus, whereas previous studies have always used a training phase, in which neutral stimuli are consistently (i.e., 100%) followed by a probe at the same location. However, previous research in other areas (e.g., obesity; alcohol abuse) applying this alternative approach (i.e., whereby about 90% of the trials are pain-incongruent) has found positive effects [26,27]. This approach has the additional advantage that it allows to investigate training changes in attention bias index without the likelihood that a post-training attention bias assessment (i.e., without training direction) dilutes ABM training effects during further test phases. The dilution of ABM training effects due to a post-training assessment has been described to be a possible reason for lacking findings in single session ABM training studies (e.g., [15]). It should however be noted that no reliable attention bias index change was found in the ABM training group. Although this finding could point at the failure of our training procedure, it is a common finding in the ABM literature. Indeed, despite changes were identified upon one or more pain-related outcomes, only two of the previous single session ABM training studies found that ABM training resulted in a reliable attention bias index change [13]. The absence of a reliable attention bias index change may therefore not per se indicate a failure of the training procedure, but be inherent to the use of the dot-probe paradigm, which does not demonstrate good reliability as a measurement tool [47]. Third, participants in the current study did not show an attention bias for pain-related information at the start of the experiment. This may have reduced chances to find an effect of ABM training [48]. Indeed, there is less room for training effects. As such, it could be argued that effects of ABM training away from pain-related information are smaller when people show no biased attention for pain-related information at baseline than when people do show a bias towards pain-related information at baseline. This finding is however not unique for the present study. A close inspection of the baseline attention bias index of previous research showing the effectiveness of a single ABM training session to modulate acute pain experience indicates that it is common that no attention bias towards pain is detected at the start of the session [10,14,15]. It is therefore unlikely that a lacking attention bias for pain-related information at baseline explains why findings differ from previous research and accordingly current null-findings.

Finally, it may also be that current null findings are because effects of ABM training do not easily translate to the experience of actual pain. Indeed, although previous studies have found that single-session ABM training affects experimental pain-related outcomes, the presented overview shows that its impact is highly variable. For example, two studies showed that ABM training away from pain stimuli increases participant’s pain threshold [10,13], while another study showed that ABM training towards affective pain stimuli increases participant’s pain threshold [15]. Future research may further aim to optimize available ABM techniques to enlarge our knowledge concerning the conditions under which ABM training has an impact upon pain-related outcomes and as such increase its impact upon real life pain-related outcomes. Increasing the number of trials or ABM sessions may be one possibility. Increasing participants’ interest will be essential in order to pursue this avenue. At current, the ABM training is monotone. More trials may make the task boring. Augmenting task interest by using motivational (e.g., a reward for good performance) or gaming elements may prove helpful [49,50]. Alternatively, ABM training techniques may also be performed within a real-life context using actual bodily sensations or cues of actual pain stimuli instead of using semantic representations of pain in a safe context [50,51]. Indeed, modifying attention bias using actual bodily sensations in the context that actually matters may increase the probability that a shift in attention bias impacts upon the experience of actual pain in this context.

Some clinical implications can cautiously be derived from the current findings. First, this study suggests that a single ABM session may be insufficient to help people cope with pain or reduce its impact upon task interference. At this time, it may be advised to use other strategies that can help people to reduce the impact of pain on task interference. Rather than directly targeting biased attention using computerized tasks, one may aim to reduce the threat value of pain, which is thought to fuel attention for pain [51]. A range of techniques is available to do so, from cognitive behavioural therapy to exposure therapy [52,53].

Some aspects of the current study require further consideration. First, there are a number of methodological differences between current study and previous studies investigating ABM training upon CPT outcomes. Therefore, this not an exact replication of previous research. For the ABM training, we opted, unlike previous ABM research in the context of pain, to include digit trials (to increase focus at the fixation cross at the beginning of each trial) and used an error message to indicate when people answered incorrect (to keep accuracy at a high level). For the CPT, we opted to raise the temperature of the cold water to reduce the intensity of the pain, i.e., from 5°C to 10°C [36]. We opted for this based on theory and previous research (see earlier). Although each of these changes is supposed to increase the reliability and impact of ABM training upon pain outcomes, further research is warranted to the exact impact of each of these changes in future ABM research. Second, participants in both groups (ABM vs sham) performed a training phase in which attentional control may have been trained. It has been argued that attentional control might be one mechanism through which ABM techniques exert their effect, rather than the direction of attentional training (e.g., [17,54]). As such, the sham training condition and ABM training condition could be equally effective, which may have masked a potential positive effect of ABM training on pain-related outcomes. Adding a third group performing a task, which does not target attentional control, may help to clarify this issue in future ABM research. Third, in both conditions (Sham and ABM), people were exposed to painful words (i.e., sensations relevant for upcoming CPT pain), which may have increased their focus for the upcoming pain. Although this is the case for all studies investigating ABM training effects on pain outcomes, and does not affect our conclusion about ABM efficacy, it may -in addition to order effects- explain why distraction was found to have no positive impact upon the pain experience. To disentangle this effect, future research may compare ABM training using non-pain information with ABM training using pain information. Fourth, participants were pain-free undergraduate students experiencing experimental pain. The homogeneity of the study sample may have limited the likelihood to find moderation effects in the follow-up analyses. Therefore, future research may want to investigate ABM effects in more heterogeneous populations to address the impact of individual difference variables upon the effectiveness of ABM training in improving pain outcomes. The inclusion of more heterogeneous groups would furthermore allow investigating the impact of ABM training, only in those people who show an attention bias at baseline or those who are highly fearful. Alternatively, future research may opt to perform ABM training in chronic pain patients, which have been found to have higher levels of pain worry as well as higher levels of attention bias for pain information [18]. Furthermore, further research is warranted to enable the generalisation of current findings to other populations. For example, a relatively small part of the participants in current sample was male. As research has shown that gender differences are important in the context of pain research (e.g., [55;56], future research should aim to include a more balanced sample. Fifth, pain ratings were done retrospectively (i.e., immediately after the CPT). Although postponed pain ratings may be susceptible to memory bias [57], post-pain ratings that are administered shortly after the exposure to pain are considered valid alternatives for online measurement [58].

## Supporting information

S1 Table Mean rating of the relevance of the sensations for the cold pressor pain. (DOCX)

S2 Table Raw dataset from this study. (XLSX)
