# Supplementary material for: Does attention bias modification training impact on task performance in the context of pain: An experimental study in healthy participants
Source: PLoS One. 2018 Jul 18;13(7):e0200629. doi: 10.1371/journal.pone.0200629 (PMC6051628; doi:10.1371/journal.pone.0200629)
Supplement: S1 Table — (DOCX) [file pone.0200629.s001.docx]

| **Pain word - Neutral word**  **[Dutch translation]** | ***Relevance of pain sensation***  ***Mean* (*SD*)** |
| --- | --- |
| Biting [bijtend] - Misty [nevelig] | 2.50 (2.60) |
| Burning [brandend] - Inarm [omhelzen] | 4.41 (2.79) |
| Chilled [verkleumd] - Assign [toewijzen] | 6.09 (2.75) |
| Cold [koud] - Meal [maal] | 8.31 (1.48) |
| Cutting [snijdend] - Assemble [monteren] | 3.64 (2.55) |
| Deaden [verdoofd] - Pet [huisdier] | 5.60 (2.40) |
| Frosty [ijzig] - Fusion [fusie] | 7.31 (1.67) |
| Gnawning [knagend] - Leister [harpoen] | 2.03 (2.03) |
| Hypothermic [onderkoeld] - Harvest month [oogstmaand] | 6.79 (2.61) |
| Itching [jeukend] - Fitting [fitting] | 0.72 (1.37) |
| Numb [gevoelloos] - Aviation [luchtvaart] | 5.24 (2.78) |
| Painful [pijnlijk] - Unconscious[onbewust] | 6.07 (2.04) |
| Pressing [drukkend] - Shopping [winkelen] | 3.67 (2.79) |
| Pulling [trekkend] - Informative [leerzaam] | 2.72 (2.53) |
| Quivering [zinderend] - Wobbling [wiebelend] | 2.98 (2.53) |
| Stabbing [stekend] - Smolder [smeulen] | 4.45 (2.63) |
| Stiffened [verstijfd] - Attempt [beproeven] | 5.81 (2.90) |
| Stinging [Prikkend] - Reedland [rietveld] | 4.40 (2.53) |
| Throbbing [kloppend] - Columns [kolommen] | 3.50 (2.61) |
| Tingling [tintelend] - Unpaid [onbetaald] | 4.60 (2.83) |
